# Supplementary material for: Early warning scores for detecting deterioration in adult hospital patients: a systematic review protocol
Source: BMJ Open. 2017 Dec 3;7(12):e019268. doi: 10.1136/bmjopen-2017-019268 (PMC5736035; doi:10.1136/bmjopen-2017-019268)
Supplement: Supplementary file 1 [file bmjopen-2017-019268supp001.pdf]

MEDLINE search strategy

Database version: Ovid MEDLINE(R) Epub Ahead of Print, In-Process & Other Non-Indexed Citations, Ovid MEDLINE(R) Daily and Ovid MEDLINE(R) 1946 to Present

1. ((National or VitalPAC or Modified or Centile or standard$) adj1 Early adj1 Warning adj1 Scor$).ti,ab.
2. (Reading adj1 Modified adj1 Early adj1 Warning adj1 Score).ti,ab.
3. (Cardiac adj1 Arrest adj1 Risk adj1 Triage).ti,ab.
4. (Assessment adj1 Score adj2 Sick adj1 patient adj1 Identification adj2 Step-up adj2 Treatment).ti,ab.
5. (Targeted adj1 Real adj1 Time adj1 Early adj1 Warning adj1 Score).ti,ab.
6. (Dutch adj1 Early adj1 Nurse adj1 Worry adj1 Indicator adj1 Score).ti,ab.
7. (Decision adj1 Tree adj1 Early adj1 Warning adj1 Score).ti,ab.
8. (Advanced adj1 Alert adj1 Monitor).ti,ab.
9. (Chronic adj1 Respiratory adj1 Early adj1 Warning adj1 Score).ti,ab.
10. (early adj1 warning adj1 (scor$ or system$)).ti,ab.
11. (track adj2 trigger adj2 (scor$ or system$)).ti,ab.
12. (physiological adj1 scoring adj1 system$).ti,ab.
13. (worry adj1 indicator adj1 scor$).ti,ab.
14. (physiological adj1 observation adj1 track adj2 trigger adj1 (scor$ or system$)).ti,ab.
15. (patient adj2 risk adj2 scoring adj1 system$).ti,ab.
16. (patient adj2 risk adj1 trigger adj1 scoring adj1 system).ti,ab.
17. (early adj1 detection adj2 patients adj2 risk).ti,ab.
18. Early Warning Score.kw.
19. Track-and-Trigger.kw.
20. "track and trigger".kw.
21. or/1-20
22. Predictive Value of Tests/
23. Monitoring, Physiologic/
24. Nursing Assessment/mt
25. Nursing Assessment/st
26. Nursing Assessment/sn
27. Severity of Illness Index/
28. Health Status Indicators/
29. Point-of-Care-Systems/

30. (NEWS or ViEWS or CART or SEWS or CREWS or PAR or PART or PSS or AAM).ti,ab.

31. OR/22-30

32. ((early or risk or warn$ or alert$ or track$ or trigger) adj2 (scor$ or system or systems)).ti,ab.

33. 31 AND 32

34. (MEWS or R-MEWS or eCART or CEWS or TREWScore or DENWIS or DTEWS or DMEWS or POTTS or PAR-T or ViEWS-L).ti,ab.

35. (develop$ or design$ or creat$ or build$ or contruct$ or validat$).ti,ab.

36. Validation Studies.pt.

37. 35 OR 36

38. 33 AND 37

39. 34 AND 37

40. 38 OR 39

41. 21 OR 40

42. ((child OR infant OR pediatrics) NOT adult).sh.

43. 41 NOT 42
