# Supplementary material for: Early warning scores for detecting deterioration in adult hospital patients: a systematic review protocol
Source: BMJ Open. 2017 Dec 3;7(12):e019268. doi: 10.1136/bmjopen-2017-019268 (PMC5736035; doi:10.1136/bmjopen-2017-019268)
Supplement: Author's manuscript [file bmjopen-2017-019268.draft_revisions.pdf]

BMJ Open is committed to open peer review. As part of this commitment we make the peer review history of every article we publish publicly available.

When an article is published we post the peer reviewers' comments and the authors' responses online. We also post the versions of the paper that were used during peer review. These are the versions that the peer review comments apply to.

The versions of the paper that follow are the versions that were submitted during the peer review process. They are not the versions of record or the final published versions. They should not be cited or distributed as the published version of this manuscript.

BMJ Open is an open access journal and the full, final, typeset and author-corrected version of record of the manuscript is available on our site with no access controls, subscription charges or pay-per-view fees (http://bmjopen.bmj.com).

If you have any questions on BMJ Open's open peer review process please email editorial.bmjopen@bmj.com

BMJ Open

Early Warning Scores for detecting deterioration in hospital patients: a systematic review protocol

Journal:	*BMJ Open*
Manuscript ID	bmjopen-2017-019268
Article Type:	Protocol
Date Submitted by the Author:	21-Aug-2017
Complete List of Authors:	Gerry, Stephen; University of Oxford, Centre for Statistics in Medicine<br>Birks, Jacqueline; University of Oxford, Centre for Statistics in Medicine<br>Bonnici, Tim; University of Oxford, UK, Nuffield Department of Medicine<br>Watkinson, Peter; Oxford University Hospitals NHS Trust, Kadoorie Centre for Critical Care research and Education<br>Kirtley, Shona; University of Oxford, Centre for Statistics in Medicine<br>Collins, Gary; University of Oxford, Centre for Statistics in Medicine
**Primary Subject Heading**:	Intensive care
Secondary Subject Heading:	Epidemiology, Research methods, Anaesthesia, Health informatics
Keywords:	Early warning scores, Development, Validation, Risk of bias

SCHOLARONE™
Manuscripts

EARLY WARNING SCORES FOR DETECTING DETERIORATION IN HOSPITAL PATIENTS: A SYSTEMATIC REVIEW PROTOCOL

Authors: Stephen Gerry, Jacqueline Birks, Timothy Bonnici, Peter Watkinson, Shona Kirtley and Gary S Collins

Corresponding author: Stephen Gerry
Address: Centre for Statistics in Medicine, Nuffield Department of Orthopaedics, Rheumatology & Musculoskeletal Sciences, University of Oxford, Windmill Road, Oxford OX3 7LD, United Kingdom. Email: Stephen.gerry@csm.ox.ac.uk. Tel: +44 (0)1865 223457

Stephen Gerry
Centre for Statistics in Medicine, Nuffield Department of Orthopaedics, Rheumatology & Musculoskeletal Sciences, University of Oxford, Windmill Road, Oxford OX3 7LD, United Kingdom

Jacqueline Birks
Centre for Statistics in Medicine, Nuffield Department of Orthopaedics, Rheumatology & Musculoskeletal Sciences, University of Oxford, Windmill Road, Oxford OX3 7LD, United Kingdom

Timothy Bonnici
Nuffield Department of Medicine, University of Oxford, John Radcliffe Hospital, Headley Way, Oxford OX3 9DU, United Kingdom

Peter Watkinson
Kadoorie Centre for Critical Care Research and Education, Nuffield Department of Clinical Neurosciences, University of Oxford, John Radcliffe Hospital, Headley Way, Oxford OX3 9DU, United Kingdom

Shona Kirtley
UK EQUATOR Centre, Centre for Statistics in Medicine, Nuffield Department of Orthopaedics, Rheumatology & Musculoskeletal Sciences, University of Oxford, Windmill Road, Oxford OX3 7LD, United Kingdom

Gary S Collins
Centre for Statistics in Medicine, Nuffield Department of Orthopaedics, Rheumatology & Musculoskeletal Sciences, University of Oxford, Windmill Road, Oxford OX3 7LD, United Kingdom

Keywords: Early warnings scores, development, validation, risk of bias

Word Count: 2414

For peer review only

Abstract

Introduction

Early warning scores (EWSs) are used extensively to identify patients at risk of deterioration in hospital. Previous systematic reviews suggest that studies which develop EWSs suffer methodological shortcomings, and consequently may fail to perform well. The reviews have also identified that few validation studies exist to test whether the scores work in other settings.

Methods

We will identify studies that describe the development or validation of EWSs. Each study will be assessed for risk of bias using the Prediction model Risk of Bias ASsessment Tool (PROBAST). Two reviewers will independently extract information. A narrative synthesis and descriptive statistics will be used to answer the main aims of the study which are to assess and critically appraise the methodological quality of the EWS, to describe the predictors included in the EWSs, and to describe the reported performance of EWSs in external validation.

Ethics and dissemination

This systematic review will only investigate published studies and therefore will not directly involve patient data. The review will help to establish whether EWSs are fit for purpose, and make recommendations to improve the quality of future research in this area.

Systematic review registration:

PROSPERO, CRD42017053324.

For peer review only

Strengths and limitations of this study

- The first systematic review in a decade to include all published Early Warning Scores.
- The first systematic review to include Early Warning Score validation studies.
- The review will assess the methodology and generalisability of studies to identify the best current Early Warning Scores, and make recommendations for future development and validation studies.
- The review will be limited to examining published Early Warning Scores. Many other scores may be in clinical use, but not published.

Background

Towards the end of the 20th century, accumulating evidence suggested that people in hospital wards were dying and suffering harm unnecessarily[1-3]. Multiple studies have demonstrated that cardiac arrest or death are commonly preceded by several hours of deranged physiology [4-6]. Recommendations were made to put systems in place to use this information to identify and respond to previously unrecognised deterioration in patients[7]. In response, the first early warning score (EWS) was published in 1997[8].

EWSs are simple tools to reduce unnecessary harm in hospitals. These clinical prediction models use patients' measured vital signs to monitor their health during their hospital stay and identify their likelihood of deteriorating, characterised as death or admission to ICU, for example. Should a patient show signs of deteriorating, the EWS triggers a warning so that care can be escalated.

There are now many EWSs available[9-11]. They are routinely used in several countries, including the Netherlands, the USA and Australia and their use in UK hospitals is mandated

For peer review only - http://bmjopen.bmj.com/site/about/guidelines.xhtml

as a standard of care by the National Institute For Health and Clinical Excellence (NICE)[12]. Based upon the he Hospital Episode Statistics[13] we estimate that EWSs are used more than 120 million times per year in the NHS in England alone, a conservative estimate that probably well underestimates the true total[*].

EWSs have been derived using a variety of approaches. Some have been developed using statistical methods for clinical prediction, by linking observations (e.g., vital signs) to outcomes (e.g., death, ICU admission) through regression models. Others have been based on clinical consensus without statistical modelling. Although there is now an abundance of clinical predication models in many fields of medicine and healthcare, in practice many of these models are scarcely used[14 15]. Systematic reviews of clinical prediction models in other clinical areas have all concluded that many are poorly developed[15-17] and that they are rarely and inappropriately evaluated[18 19] (often referred to as validation). There is no common agreement on which of the dozens of EWSs available performs best. Most problematically, recent evidence suggests that EWSs have not solved the problem they were designed for: unrecognised deterioration of patients in hospitals remains a major issue[20].

The aim of this systematic review is to critically appraise papers describing the development and validation of EWSs, with a particular focus on methodology, reporting and generalisability, in order to identify high quality EWSs and provide guidance regarding the methods to develop and validate future EWSs.

[*] (~12 million non-day-cases per year * mean length of stay 5 days * 2 observations per day)

For peer review only - http://bmjopen.bmj.com/site/about/guidelines.xhtml

Existing systematic reviews

Three systematic reviews on EWSs have been published[9-11]. Those by Gao et al. (2007)[9] and GB Smith et al. (2008)[10] were published almost a decade ago, whilst MEB Smith et al. (2014) used narrow inclusion criteria and did not include all available EWSs[11]. Several new EWSs have been published since.

The main aims of the reviews were to describe the development of EWSs, assess their predictive performance, and assess any impact studies that evaluate the effect of implementing EWSs in clinical practice. A fourth review by Alam[21] solely looked at impact studies, but we do not plan to include these in our review.

Many of the reviewed scores included similar predictors and applied similar weights to those predictors. Nearly all of the scores included pulse rate, breathing rate, systolic blood pressure, and temperature. The reviews also found some indication that scores that included age performed better[10]. In contrast to studies developing EWSs, validation studies that evaluated the performance of EWSs were relatively uncommon.

The use of poor methods to develop EWSs could mean that the scores are unreliable and fail to accurately predict risk. Gao et al. (2007) and MEB Smith et al. (2014) subjectively reported that they found many of the primary studies to be of low quality, used suboptimal methods, and were at high risk of bias[9 11]. However, none of the reviews made a detailed and structured evaluation of the approaches used to develop EWSs, following recommended methodological considerations in the field of clinical prediction models[22-26].

For peer review only - http://bmjopen.bmj.com/site/about/guidelines.xhtml

After a prediction model (i.e., an EWS) has been developed, its predictive accuracy should be evaluated in the same population used to derive it, a process called internal validation. The two widely recommended characteristics that describe the performance of a prediction model are discrimination (e.g., the c-index and AUROC) and calibration[22]. Discrimination reflects a prediction model's ability to differentiate between those who develop an outcome (i.e., death) and those who do not. A model should predict higher risks for those who develop the outcome. Calibration reflects the level of agreement between observed outcomes and the model's predictions.

Both discrimination and calibration must be assessed and reported to judge a model's accuracy[22]. However, as in many other clinical areas, studies evaluating EWSs have tended to give more prominence to discrimination and have rarely assessed model calibration. Two of the reviews investigated how primary EWS studies report predictive performance, with conflicting conclusions. Gao et al. (2007) found unacceptance predictive performance[9], whereas MEB Smith et al. (2014)[11] found good predictive performance. This difference in result may reflect differences in the included studies and how the authors assessed model performance.

Internal validation provides insights into model performance in the same population used to derive the model. In contrast, external validation assesses the model's performance in a different population from that used to derive it. External validation assesses model discrimination and calibration to determine whether the model performs satisfactorily in data other than that it was developed with, which is called generalisability[27]. Although the three reviews did not include or focus on external validation studies, they all highlighted a lack of external validation studies of EWSs. GB Smith et al. (2008) did not investigate validation

For peer review only - http://bmjopen.bmj.com/site/about/guidelines.xhtml

For peer review only

studies, but performed their own external validation as part of their review by evaluating the identified models using their own data. They found that none of the scores showed good enough performance[10].

Research aims

In this systematic review, we aim to identify all existing published EWSs and:

1. Describe and critically appraise the methods that have been used to develop and validate (where appropriate) the scores. We will take a wide-ranging approach and will cover statistical aspects, such as how missing data are accounted for and how continuous predictors are used. We will also investigate aspects of generalisability, such as details of the populations used to develop the models.
2. Describe which predictors are included in the scores and how they are weighted.
3. Report which EWSs have undergone external validation and, if so, how well they performed.

Methods

Our systematic review protocol was registered with the International Prospective Register of Systematic Reviews (PROSPERO) on 12th July 2017 (registration number CRD42017053324). Our systematic review will be carried out and reported in accordance with two published guidelines: the Critical Appraisal and Data Extraction for Systematic Reviews of Prediction Modelling Studies (CHARMS) checklist[28] and the Preferred Reporting Items for Systematic Reviews and Meta-Analyses (PRISMA) checklist[29].

For peer review only

For peer review only - http://bmjopen.bmj.com/site/about/guidelines.xhtml

Selection criteria

We will include studies that satisfy all of the following criteria:

1. The study describes the development or validation of one or more EWSs, defined as a score used to identify hospitalised patients at risk of clinical deterioration.
2. The EWS studied combines information from at least two predictor variables to produce a summary risk estimate.
3. Validation studies will only be included where the corresponding development articles are available.

We will exclude papers where any of the following apply:

1. The score was developed for use in a subset of patients with a specific disease or group of diseases.
2. The score was developed for use with children (aged under 16 years) or pregnant women.
3. The score is intended for outpatient use.
4. Reviews, letters, personal correspondence and abstracts.

Search strategy

Studies will be identified by searching the medical literature using Medline (OVID), CINAHL (EbscoHost), and Embase (OVID) to identify primary articles reporting on the development and/or validation of EWSs. We will use a combination of relevant controlled vocabulary terms for each database (e.g. MeSH, Emtree), and free-text search terms. Citation lists of previous systematic reviews and included studies will be searched to identify any

For peer review only - http://bmjopen.bmj.com/site/about/guidelines.xhtml

For peer review only

studies missed by the search. We will also conduct a Google Scholar search to identify any other eligible studies. Appendix A shows a draft search strategy.

Study selection

Two reviewers will independently screen all titles and abstracts using pre-specified screening criteria. The full text of any relevant articles will then be independently assessed by two reviewers. Disagreements will be resolved by discussion and, if necessary, referral to a third reviewer. The study selection process will be reported using a PRISMA flow diagram[29].

Data extraction

Data will be independently extracted by two reviewers using a standardised and piloted data extraction form. The form will be administered using the Research Electronic Data Capture (REDCap) electronic data capture tool[30]. Disagreements will be resolved by discussion and, if necessary, by referral to a third reviewer. We will choose items for extraction based on the CHARMS checklist[28], supplemented by subject-specific questions and methodological guidance. Items for extraction will include:

- Study characteristics [development and validation] (e.g., country, year)
- Study design [development and validation] (e.g., prospective, case control, cohort, clinical consensus)
- Patient characteristics [development and validation] (e.g., hospital ward, age, sex)
- Predicted outcome [development and validation] (e.g., survival at 24 hours, ICU admission at 24 hours)

For peer review only - http://bmjopen.bmj.com/site/about/guidelines.xhtml

For peer review only

- Model development [development] (e.g., sample size, type of model, handling of continuous variables, selection of variables, missing data, method of internal validation)
- Model presentation [development] (e.g., full regression model, simplified model, risk groups)
- Assessment of performance [development and validation] (e.g., measures of discrimination, measures of calibration)

Assessment of bias

Each article will be independently assessed by two reviewers using the Prediction model Risk of Bias ASsessment Tool (PROBAST), which was recently developed by the Cochrane Prognosis Methods Group to assess the quality and risk of bias for prediction models (due to be submitted shortly; Wolff R, Whiting, Mallett S *et al. [including author GSC]*, personal communication). PROBAST consists of 23 signalling questions within four domains (participant selection, predictors, outcome, and analysis).

Evidence synthesis

We will summarise the results using descriptive statistics, graphical plots, and a narrative synthesis. We do not plan to perform a quantitative synthesis of the scores or their predictive performance. However, if we identify multiple studies that evaluate the same EWS and report common performance measures, we will summarise their performance using a random-effects meta-analysis[31]. The PROBAST evaluation will be used to determine the models' risk of bias, including whether the EWSs are likely to work as intended for the hospital population of interest. The models will be classed as low, high, or unclear risk of bias.

For peer review only - http://bmjopen.bmj.com/site/about/guidelines.xhtml

Discussion

Although EWSs are extensively used in clinical practice, the methodology behind them remains questionable. Although not formally assessed, previous systematic reviews of EWSs have indicated that many studies suffer from a lack of quality and that few EWSs have been satisfactorily validated[9-11]. These aspects are crucial for developing a prediction model that can confidently be rolled out into clinical practice. This systematic review will bridge this important gap by examining methodological quality and external validation in detail. This systematic review is timely, as it is now nearly a decade since the last comprehensive review of EWSs, which have only existed for 20 years.

EWSs have historically been implemented as part of traditional paper observation charts. The requirement for scores to be calculated manually necessitated the use of simple scoring algorithms. Storage of data on paper has been a barrier to collection of large datasets for score derivation and validation. Digital systems are increasingly being used to record vital signs and calculate EWSs[32], offering the opportunity to be more rigorous and innovative in the development and implementation of new EWSs. The adoption of digital vital signs charting offers an opportunity to transition away from poor quality EWSs. Our review will provide the evidence for creators of digital systems to identify which EWSs should be prioritised for implementation.

Contributors

SG, JB, TB, PW, and GSC conceived the study. SG developed the study protocol and will implement the systematic review under the supervision of GSC. SG will provide the study's statistical analysis plan and will analyse the data. SG and SK will perform the study search and SG will screen and extract the data. JB, TB, PW, and GSC will review the work. SG

For peer review only - http://bmjopen.bmj.com/site/about/guidelines.xhtml

wrote the first protocol manuscript draft and all authors gave input into and approved the final draft of the protocol.

Funding

SG is funded by an NIHR Doctoral Fellowship (DRF-2016-09-073). JB, TB, PW and GSC are supported by the NIHR Biomedical Research Centre, Oxford. The funders have not played any role in the development of this protocol.

Competing interests

None declared

Data sharing statement

All unpublished data will be made available on request. The first author, SG, should be contacted with requests for unpublished data.

References

1. Brennan TA, Leape LL, Laird NM, et al. Incidence of Adverse Events and Negligence in Hospitalized Patients. *New England Journal of Medicine* 1991;324(6):370-76. doi: 10.1056/nejm199102073240604
2. Institute of Medicine Committee on Quality of Health Care in A. In: Kohn LT, Corrigan JM, Donaldson MS, eds. To Err is Human: Building a Safer Health System. Washington (DC): National Academies Press (US) 2000.
3. Vincent C, Neale G, Woloshynowych M. Adverse events in British hospitals: preliminary retrospective record review. *BMJ* 2001;322(7285):517-19. doi: 10.1136/bmj.322.7285.517
4. Hillman KM, Bristow PJ, Chey T, et al. Duration of life-threatening antecedents prior to intensive care admission. *Intensive Care Med* 2002;28(11):1629-34. doi: 10.1007/s00134-002-1496-y [published Online First: 2002/11/05]
5. Kause J, Smith G, Prytherch D, et al. A comparison of antecedents to cardiac arrests, deaths and emergency intensive care admissions in Australia and New Zealand, and the United Kingdom--the ACADEMIA study. *Resuscitation* 2004;62(3):275-82. doi: 10.1016/j.resuscitation.2004.05.016 [published Online First: 2004/08/25]
6. Hogan H, Healey F, Neale G, et al. Preventable deaths due to problems in care in English acute hospitals: a retrospective case record review study. *BMJ Quality & Safety* 2012

For peer review only - http://bmjopen.bmj.com/site/about/guidelines.xhtml

7. McQuillan P, Pilkington S, Allan A, et al. Confidential inquiry into quality of care before admission to intensive care. *Bmj* 1998;316(7148):1853-8. [published Online First: 1998/06/19]
8. Morgan RJM, Williams F, Wright MM. An early warning scoring system for detecting developing critical illness. *Clin Intensive Care* 1997;8:100.
9. Gao H, McDonnell A, Harrison DA, et al. Systematic review and evaluation of physiological track and trigger warning systems for identifying at-risk patients on the ward. *Intensive Care Medicine* 2007;33(4):667-79. doi: 10.1007/s00134-007-0532-3
10. Smith GB, Prytherch DR, Schmidt PE, et al. Review and performance evaluation of aggregate weighted 'track and trigger' systems. *Resuscitation* 2008;77(2):170-79. doi: 10.1016/j.resuscitation.2007.12.004
11. Smith MEB, Chiovaro JC, O'Neil M, et al. Early Warning System Scores for Clinical Deterioration in Hospitalized Patients: A Systematic Review. *Annals of the American Thoracic Society* 2014;11(9):1454-65. doi: 10.1513/AnnalsATS.201403-102OC
12. Excellence NIfHaC. Acutely ill adults in hospital: recognising and responding to deterioration. (NICE guideline CG50). 2007
13. Centre HSCI. Hospital Episode Statistics. Admitted Patient Care, England - 2014-15. 2015
14. Damen JAAG, Hooft L, Schuit E, et al. Prediction models for cardiovascular disease risk in the general population: systematic review. *BMJ* 2016;353 doi: 10.1136/bmj.i2416
15. Kleinrouweler CE, Cheong-See FM, Collins GS, et al. Prognostic models in obstetrics: available, but far from applicable. *Am J Obstet Gynecol* 2016;214(1):79-90.e36. doi: 10.1016/j.ajog.2015.06.013 [published Online First: 2015/06/14]
16. Bouwmeester W, Zuithoff NP, Mallett S, et al. Reporting and methods in clinical prediction research: a systematic review. *PLoS Med* 2012;9(5):1-12. doi: 10.1371/journal.pmed.1001221 [published Online First: 2012/05/26]
17. Collins GS, Mallett S, Omar O, et al. Developing risk prediction models for type 2 diabetes: a systematic review of methodology and reporting. *BMC Med* 2011;9:103. doi: 10.1186/1741-7015-9-103 [published Online First: 2011/09/10]
18. Collins GS, de Groot JA, Dutton S, et al. External validation of multivariable prediction models: a systematic review of methodological conduct and reporting. *BMC Med Res Methodol* 2014;14:40. doi: 10.1186/1471-2288-14-40 [published Online First: 2014/03/22]
19. Mallett S, Royston P, Waters R, et al. Reporting performance of prognostic models in cancer: a review. *BMC Med* 2010;8:21. doi: 10.1186/1741-7015-8-21 [published Online First: 2010/04/01]
20. NCEPOD. Time to Intervene? A review of patients who underwent cardiopulmonary rescuscitation as a result of an in-hospital cardiorespiratory arrest. 2012
21. Alam N, Hobbelink EL, van Tienhoven AJ, et al. The impact of the use of the Early Warning Score (EWS) on patient outcomes: a systematic review. *Resuscitation* 2014;85(5):587-94. doi: 10.1016/j.resuscitation.2014.01.013 [published Online First: 2014/01/29]
22. Collins GS, Reitsma JB, Altman DG, et al. Transparent reporting of a multivariable prediction model for individual prognosis or diagnosis (tripod): The tripod statement. *Annals of Internal Medicine* 2015;162(1):55-63. doi: 10.7326/M14-0697
23. Moons KG, Altman DG, Reitsma JB, et al. Transparent Reporting of a multivariable prediction model for Individual Prognosis or Diagnosis (TRIPOD): explanation and elaboration. *Ann Intern Med* 2015;162(1):W1-73. doi: 10.7326/m14-0698 [published Online First: 2015/01/07]
24. Moons KG, Kengne AP, Woodward M, et al. Risk prediction models: I. Development, internal validation, and assessing the incremental value of a new (bio)marker. *Heart* 2012;98(9):683-90. doi: 10.1136/heartjnl-2011-301246 [published Online First: 2012/03/09]
25. Steyerberg EW, Moons KG, van der Windt DA, et al. Prognosis Research Strategy (PROGRESS) 3: prognostic model research. *PLoS Med* 2013;10(2):e1001381. doi: 10.1371/journal.pmed.1001381 [published Online First: 2013/02/09]

For peer review only

26. Steyerberg EW, Vergouwe Y. Towards better clinical prediction models: seven steps for development and an ABCD for validation. *Eur Heart J* 2014;35(29):1925-31. doi: 10.1093/eurheartj/ehu207 [published Online First: 2014/06/06]
27. Justice AC, Covinsky KE, Berlin JA. Assessing the generalizability of prognostic information. *Ann Intern Med* 1999;130(6):515-24. [published Online First: 1999/03/13]
28. Moons KG, de Groot JA, Bouwmeester W, et al. Critical appraisal and data extraction for systematic reviews of prediction modelling studies: the CHARMS checklist. *PLoS Med* 2014;11(10):e1001744. doi: 10.1371/journal.pmed.1001744 [published Online First: 2014/10/15]
29. Moher D, Liberati A, Tetzlaff J, et al. Preferred Reporting Items for Systematic Reviews and Meta-Analyses: The PRISMA Statement. *PLOS Medicine* 2009;6(7):e1000097. doi: 10.1371/journal.pmed.1000097
30. Harris PA, Taylor R, Thielke R, et al. Research Electronic Data Capture (REDCap) - A metadata-driven methodology and workflow process for providing translational research informatics support. *Journal of biomedical informatics* 2009;42(2):377-81. doi: 10.1016/j.jbi.2008.08.010
31. Debray TP, Damen JA, Snell KI, et al. A guide to systematic review and meta-analysis of prediction model performance. *Bmj* 2017;356:i6460. doi: 10.1136/bmj.i6460 [published Online First: 2017/01/07]
32. Wong D, Bonnici T, Knight J, et al. SEND: a system for electronic notification and documentation of vital sign observations. *BMC Medical Informatics and Decision Making* 2015;15:68. doi: 10.1186/s12911-015-0186-y

For peer review only

For peer review only - http://bmjopen.bmj.com/site/about/guidelines.xhtml

MEDLINE search strategy

Database version: Ovid MEDLINE(R) Epub Ahead of Print, In-Process & Other Non-Indexed Citations, Ovid MEDLINE(R) Daily and Ovid MEDLINE(R) 1946 to Present

1. ((National or VitalPAC or Modified or Centile or standard$) adj1 Early adj1 Warning adj1 Scor$).ti,ab.
2. (Reading adj1 Modified adj1 Early adj1 Warning adj1 Score).ti,ab.
3. (Cardiac adj1 Arrest adj1 Risk adj1 Triage).ti,ab.
4. (Assessment adj1 Score adj2 Sick adj1 patient adj1 Identification adj2 Step-up adj2 Treatment).ti,ab.
5. (Targeted adj1 Real adj1 Time adj1 Early adj1 Warning adj1 Score).ti,ab.
6. (Dutch adj1 Early adj1 Nurse adj1 Worry adj1 Indicator adj1 Score).ti,ab.
7. (Decision adj1 Tree adj1 Early adj1 Warning adj1 Score).ti,ab.
8. (Advanced adj1 Alert adj1 Monitor).ti,ab.
9. (Chronic adj1 Respiratory adj1 Early adj1 Warning adj1 Score).ti,ab.
10. (early adj1 warning adj1 (scor$ or system$)).ti,ab.
11. (track adj2 trigger adj2 (scor$ or system$)).ti,ab.
12. (physiological adj1 scoring adj1 system$).ti,ab.
13. (worry adj1 indicator adj1 scor$).ti,ab.
14. (physiological adj1 observation adj1 track adj2 trigger adj1 (scor$ or system$)).ti,ab.
15. (patient adj2 risk adj2 scoring adj1 system$).ti,ab.
16. (patient adj2 risk adj1 trigger adj1 scoring adj1 system).ti,ab.
17. (early adj1 detection adj2 patients adj2 risk).ti,ab.
18. Early Warning Score.kw.
19. Track-and-Trigger.kw.
20. "track and trigger".kw.
21. or/1-20
22. Predictive Value of Tests/
23. Monitoring, Physiologic/
24. Nursing Assessment/mt
25. Nursing Assessment/st
26. Nursing Assessment/sn
27. Severity of Illness Index/
28. Health Status Indicators/
29. Point-of-Care-Systems/

For peer review only

30. (NEWS or ViEWS or CART or SEWS or CREWS or PAR or PART or PSS or AAM).ti,ab.

31. OR/22-30

32. ((early or risk or warn$ or alert$ or track$ or trigger) adj2 (scor$ or system or systems)).ti,ab.

33. 31 AND 32

34. (MEWS or R-MEWS or eCART or CEWS or TREWScore or DENWIS or DTEWS or DMEWS or POTTS or PAR-T or ViEWS-L).ti,ab.

35. (develop$ or design$ or creat$ or build$ or contruct$ or validat$).ti,ab.

36. Validation Studies.pt.

37. 35 OR 36

38. 33 AND 37

39. 34 AND 37

40. 38 OR 39

41. 21 OR 40

42. ((child OR infant OR pediatrics) NOT adult).sh.

43. 41 NOT 42

For peer review only

1

PRISMA-P 2015 Checklist

Section/topic	#	Checklist item	Information reported Yes	Information reported No	Page number
**ADMINISTRATIVE INFORMATION**					
**Title**					
Identification	1a	Identify the report as a protocol of a systematic review	☒	☐	1
Update	1b	If the protocol is for an update of a previous systematic review, identify as such	☒	☐	NA
**Registration**	2	If registered, provide the name of the registry (e.g., PROSPERO) and registration number in the Abstract	☒	☐	2 & 7
**Authors**					
Contact	3a	Provide name, institutional affiliation, and e-mail address of all protocol authors; provide physical mailing address of corresponding author	☒	☐	1
Contributions	3b	Describe contributions of protocol authors and identify the guarantor of the review	☒	☐	11 & 12
**Amendments**	4	If the protocol represents an amendment of a previously completed or published protocol, identify as such and list changes; otherwise, state plan for documenting important protocol amendments	☒	☐	NA
**Support**					
Sources	5a	Indicate sources of financial or other support for the review	☒	☐	12
Sponsor	5b	Provide name for the review funder and/or sponsor	☒	☐	12
Role of sponsor/funder	5c	Describe roles of funder(s), sponsor(s), and/or institution(s), if any, in developing the protocol	☒	☐	12
**INTRODUCTION**					
**Rationale**	6	Describe the rationale for the review in the context of what is already known	☒	☐	3 & 4
**Objectives**	7	Provide an explicit statement of the question(s) the review will address with reference to participants, interventions, comparators, and outcomes (PICO)	☒	☐	7
**METHODS**					
**Eligibility criteria**	8	Specify the study characteristics (e.g., PICO, study design, setting, time frame) and report characteristics (e.g., years considered, language, publication status) to be used as criteria for eligibility for the review	☒	☐	8

For peer review only

Section/topic	#	Checklist item	Information reported		Page number
			Yes	No	
**Information sources**	9	Describe all intended information sources (e.g., electronic databases, contact with study authors, trial registers, or other grey literature sources) with planned dates of coverage	☒	☐	8
**Search strategy**	10	Present draft of search strategy to be used for at least one electronic database, including planned limits, such that it could be repeated	☒	☐	Page 15 (Appendix A)
STUDY RECORDS					
Data management	11a	Describe the mechanism(s) that will be used to manage records and data throughout the review	☒	☐	9
Selection process	11b	State the process that will be used for selecting studies (e.g., two independent reviewers) through each phase of the review (i.e., screening, eligibility, and inclusion in meta-analysis)	☒	☐	9
Data collection process	11c	Describe planned method of extracting data from reports (e.g., piloting forms, done independently, in duplicate), any processes for obtaining and confirming data from investigators	☒	☐	9
**Data items**	12	List and define all variables for which data will be sought (e.g., PICO items, funding sources), any pre-planned data assumptions and simplifications	☒	☐	9
**Outcomes and prioritization**	13	List and define all outcomes for which data will be sought, including prioritization of main and additional outcomes, with rationale	☒	☐	9
**Risk of bias in individual studies**	14	Describe anticipated methods for assessing risk of bias of individual studies, including whether this will be done at the outcome or study level, or both; state how this information will be used in data synthesis	☒	☐	10
DATA					
**Synthesis**	15a	Describe criteria under which study data will be quantitatively synthesized	☒	☐	10
	15b	If data are appropriate for quantitative synthesis, describe planned summary measures, methods of handling data, and methods of combining data from studies, including any planned exploration of consistency (e.g., I^2, Kendall's tau)	☒	☐	10
	15c	Describe any proposed additional analyses (e.g., sensitivity or subgroup analyses, meta-regression)	☒	☐	NA
	15d	If quantitative synthesis is not appropriate, describe the type of summary planned	☒	☐	10
**Meta-bias(es)**	16	Specify any planned assessment of meta-bias(es) (e.g., publication bias across studies, selective reporting within studies)	☒	☐	NA
**Confidence in cumulative evidence**	17	Describe how the strength of the body of evidence will be assessed (e.g., GRADE)	☒	☐	NA

For peer review only

BMJ Open

Early Warning Scores for detecting deterioration in adult hospital patients: a systematic review protocol

Journal:	*BMJ Open*
Manuscript ID	bmjopen-2017-019268.R1
Article Type:	Protocol
Date Submitted by the Author:	28-Sep-2017
Complete List of Authors:	Gerry, Stephen; University of Oxford, Centre for Statistics in Medicine<br>Birks, Jacqueline; University of Oxford, Centre for Statistics in Medicine<br>Bonnici, Tim; University of Oxford, UK, Nuffield Department of Medicine<br>Watkinson, Peter; Oxford University Hospitals NHS Trust, Kadoorie Centre for Critical Care research and Education<br>Kirtley, Shona; University of Oxford, Centre for Statistics in Medicine<br>Collins, Gary; University of Oxford, Centre for Statistics in Medicine
**Primary Subject Heading**:	Intensive care
Secondary Subject Heading:	Epidemiology, Research methods, Anaesthesia, Health informatics
Keywords:	Early warning scores, Development, Validation, Risk of bias

SCHOLARONE™
Manuscripts

For peer review only

EARLY WARNING SCORES FOR DETECTING DETERIORATION IN ADULT HOSPITAL PATIENTS: A SYSTEMATIC REVIEW PROTOCOL

Authors: Stephen Gerry, Jacqueline Birks, Timothy Bonnici, Peter Watkinson, Shona Kirtley and Gary S Collins

Corresponding author: Stephen Gerry
Address: Centre for Statistics in Medicine, Nuffield Department of Orthopaedics, Rheumatology & Musculoskeletal Sciences, University of Oxford, Windmill Road, Oxford OX3 7LD, United Kingdom. Email: Stephen.gerry@csm.ox.ac.uk. Tel: +44 (0)1865 223457

Stephen Gerry
Centre for Statistics in Medicine, Nuffield Department of Orthopaedics, Rheumatology & Musculoskeletal Sciences, University of Oxford, Windmill Road, Oxford OX3 7LD, United Kingdom

Jacqueline Birks
Centre for Statistics in Medicine, Nuffield Department of Orthopaedics, Rheumatology & Musculoskeletal Sciences, University of Oxford, Windmill Road, Oxford OX3 7LD, United Kingdom

Timothy Bonnici
Nuffield Department of Medicine, University of Oxford, John Radcliffe Hospital, Headley Way, Oxford OX3 9DU, United Kingdom

Peter Watkinson
Kadoorie Centre for Critical Care Research and Education, Nuffield Department of Clinical Neurosciences, University of Oxford, John Radcliffe Hospital, Headley Way, Oxford OX3 9DU, United Kingdom

Shona Kirtley
UK EQUATOR Centre, Centre for Statistics in Medicine, Nuffield Department of Orthopaedics, Rheumatology & Musculoskeletal Sciences, University of Oxford, Windmill Road, Oxford OX3 7LD, United Kingdom

Gary S Collins
Centre for Statistics in Medicine, Nuffield Department of Orthopaedics, Rheumatology & Musculoskeletal Sciences, University of Oxford, Windmill Road, Oxford OX3 7LD, United Kingdom

Keywords: Early warnings scores, early warning systems, development, validation, risk of bias

Word Count: 2571

For peer review only - http://bmjopen.bmj.com/site/about/guidelines.xhtml

For peer review only

Abstract

Introduction

Early warning scores (EWSs) are used extensively to identify patients at risk of deterioration in hospital. Previous systematic reviews suggest that studies which develop EWSs suffer methodological shortcomings, and consequently may fail to perform well. The reviews have also identified few validation studies exist to test whether the scores work in other settings. We will aim to systematically review papers describing the development or validation of EWSs, focusing on methodology, generalisability and reporting.

Methods

We will identify studies that describe the development or validation of EWSs for adult hospital inpatients. Each study will be assessed for risk of bias using the Prediction model Risk of Bias ASsessment Tool (PROBAST). Two reviewers will independently extract information. A narrative synthesis and descriptive statistics will be used to answer the main aims of the study which are to assess and critically appraise the methodological quality of the EWS, to describe the predictors included in the EWSs, and to describe the reported performance of EWSs in external validation.

Ethics and dissemination

This systematic review will only investigate published studies and therefore will not directly involve patient data. The review will help to establish whether EWSs are fit for purpose, and make recommendations to improve the quality of future research in this area.

Systematic review registration:

PROSPERO, CRD42017053324.

For peer review only

Strengths and limitations of this study

- The first systematic review in a decade to include all published Early Warning Scores.
- The first systematic review to include Early Warning Score validation studies.
- The review will assess the methodology and generalisability of studies to identify the best current Early Warning Scores, and make recommendations for future development and validation studies.
- The review will be limited to examining published Early Warning Scores. Many other scores may be in clinical use, but not published.

Background

Towards the end of the 20th century, accumulating evidence suggested that people in hospital wards were dying and suffering harm unnecessarily[1-3]. Multiple studies have demonstrated that cardiac arrest or death are commonly preceded by several hours of deranged physiology [4-6]. Recommendations were made to put systems in place to use this information to identify and respond to previously unrecognised deterioration in patients[7]. In response, the first early warning score (EWS) was published in 1997[8].

EWSs are simple tools to reduce unnecessary harm in hospitals. These clinical prediction models use patients' measured vital signs to monitor their health during their hospital stay and identify their likelihood of deteriorating, characterised as death or admission to ICU, for example. Should a patient show signs of deteriorating, the EWS triggers a warning so that care can be escalated. EWSs, which are also commonly referred to as track-and-trigger scores, are often implemented as part of an 'early warning system', or 'early warning score system'. These are computer systems which record vital signs, automatically or manually, and then implement the EWS algorithm to indicate a patient's risk of deterioration. The

For peer review only - http://bmjopen.bmj.com/site/about/guidelines.xhtml

interest of this review lies in the underlying scoring systems/algorithms themselves, and not the systems in which they are implemented.

There are now many EWSs available[9-11]. They are routinely used in several countries, including the Netherlands, the USA and Australia and their use in UK hospitals is mandated as a standard of care by the National Institute For Health and Clinical Excellence (NICE)[12]. Based upon the Hospital Episode Statistics[13] we estimate that EWSs are used more than 120 million times per year in the NHS in England alone, a conservative estimate that probably well underestimates the true total[*].

EWSs have been derived using a variety of approaches. Some have been developed using statistical methods for clinical prediction, by linking observations (e.g., vital signs) to outcomes (e.g., death, ICU admission) through regression models. Others have been based on clinical consensus without statistical modelling. Although there is now an abundance of clinical predication models in many fields of medicine and healthcare, in practice many of these models are scarcely used[14 15]. Systematic reviews of clinical prediction models in other clinical areas have all concluded that many are poorly developed[15-17] and that they are rarely and inappropriately evaluated[18 19] (often referred to as validation), i.e. tested in different setting to which they were developed. There is no common agreement on which of the dozens of EWSs available performs best. Most problematically, recent evidence suggests that EWSs have not solved the problem they were designed for: unrecognised deterioration of patients in hospitals remains a major issue[20].

[*] (~12 million non-day-cases per year * mean length of stay 5 days * 2 observations per day)

For peer review only - http://bmjopen.bmj.com/site/about/guidelines.xhtml

The aim of this systematic review is to critically appraise papers describing the development and validation of EWSs for adult hospital inpatients, with a particular focus on methodology, reporting and generalisability, in order to identify high quality EWSs and provide guidance regarding the methods to develop and validate future EWSs.

Existing systematic reviews

Four systematic reviews of studies which develop or validate EWSs have been published[9-11]. Those by Gao et al. (2007)[9] and GB Smith et al. (2008)[10] were published almost a decade ago, whilst MEB Smith et al. (2014) used narrow inclusion criteria and did not include all available EWSs[11], and the review by Kyriacos et al (2011)[21] was a more general overview of the literature. Several new EWSs have been published since.

The main aims of the reviews were to describe the development of EWSs, assess their predictive performance, and assess any impact studies that evaluate the effect of implementing EWSs in clinical practice. Other reviews, such as those by Alam[22] and McGaughey[23], looked at impact studies, but we do not plan to include these in our review.

Many of the reviewed scores included similar predictors and applied similar weights to those predictors. Nearly all of the scores included pulse rate, breathing rate, systolic blood pressure, and temperature. The reviews also found some indication that scores that included age performed better[10]. In contrast to studies developing EWSs, validation studies that evaluated the performance of EWSs were relatively uncommon.

The use of poor methods to develop EWSs could mean that the scores are unreliable and fail to accurately predict risk. Gao et al. (2007) and MEB Smith et al. (2014) subjectively

For peer review only - http://bmjopen.bmj.com/site/about/guidelines.xhtml

reported that they found many of the primary studies to be of low quality, used suboptimal methods, and were at high risk of bias[9 11]. However, none of the reviews made a detailed and structured evaluation of the approaches used to develop EWSs, following recommended methodological considerations in the field of clinical prediction models[24-28].

After a prediction model (i.e., an EWS) has been developed, its predictive accuracy should be evaluated in the same population used to derive it, a process called internal validation. The two widely recommended characteristics that describe the performance of a prediction model are discrimination (e.g., the c-index and AUROC) and calibration[24]. Discrimination reflects a prediction model's ability to differentiate between those who develop an outcome (i.e., death) and those who do not. A model should predict higher risks for those who develop the outcome. Calibration reflects the level of agreement between observed outcomes and the model's predictions.

Both discrimination and calibration must be assessed and reported to judge a model's accuracy[24]. However, as in many other clinical areas, studies evaluating EWSs have tended to give more prominence to discrimination and have rarely assessed model calibration. Two of the reviews investigated how primary EWS studies report predictive performance, with conflicting conclusions. Gao et al. (2007) found unacceptance predictive performance[9], whereas MEB Smith et al. (2014)[11] found good predictive performance. This difference in result may reflect differences in the included studies and how the authors assessed model performance.

Internal validation provides insights into model performance in the same population used to derive the model. In contrast, external validation assesses the model's performance in a

For peer review only - http://bmjopen.bmj.com/site/about/guidelines.xhtml

different population from that used to derive it. External validation assesses model discrimination and calibration to determine whether the model performs satisfactorily in data other than that it was developed with, which is called generalisability[29]. Although the four reviews did not have a specific focus on external validation studies, they all highlighted a lack of external validation studies of EWSs. GB Smith et al. (2008) did not investigate validation studies, but performed their own external validation as part of their review by evaluating the identified models using their own data. They found that none of the scores showed good enough performance[10].

Research aims

In this systematic review, we aim to identify all existing published EWSs for adult hospital inpatients and:

1. Describe and critically appraise the methods that have been used to develop and validate (where appropriate) the scores. We will take a wide-ranging approach and will cover statistical aspects, such as how missing data are accounted for and how continuous predictors are used. We will also investigate aspects of generalisability, such as details of the populations used to develop the models.
2. Describe which predictors are included in the scores and how they are weighted.
3. Report which EWSs have undergone external validation and, if so, how well they performed.

Methods

Our systematic review protocol was registered with the International Prospective Register of Systematic Reviews (PROSPERO) on 12th July 2017 (registration number

For peer review only - http://bmjopen.bmj.com/site/about/guidelines.xhtml

For peer review only

CRD42017053324). Our systematic review will be carried out and reported in accordance with two published guidelines: the Critical Appraisal and Data Extraction for Systematic Reviews of Prediction Modelling Studies (CHARMS) checklist[30] and the Preferred Reporting Items for Systematic Reviews and Meta-Analyses (PRISMA) checklist[31].

Selection criteria

We will include studies that satisfy all of the following criteria:

1. The study describes the development or validation of one or more EWSs, defined as a score used to identify hospitalised patients at risk of clinical deterioration.
2. The EWS studied combines information from at least two predictor variables to produce a summary risk estimate.
3. Validation studies will only be included where the corresponding development articles are available.

We will exclude papers where any of the following apply:

1. The score was developed for use in a subset of patients with a specific disease or group of diseases.
2. The score was developed for use with children (aged under 16 years) or pregnant women.
3. The score is intended for outpatient use.
4. The score is intended for use in the intensive care unit (ICU).
5. Reviews, letters, personal correspondence and abstracts.

For peer review only

For peer review only - http://bmjopen.bmj.com/site/about/guidelines.xhtml

Search strategy

Studies will be identified by searching the medical literature using Medline (OVID), CINAHL (EbscoHost), and Embase (OVID) to identify primary articles reporting on the development and/or validation of EWSs. We will use a combination of relevant controlled vocabulary terms for each database (e.g. MeSH, Emtree), and free-text search terms. No date or language restrictions will be applied. Citation lists of previous systematic reviews and included studies will be searched to identify any studies missed by the search. We will also conduct a Google Scholar search to identify any other eligible studies. Appendix A shows a draft search strategy.

Study selection

Two reviewers will independently screen all titles and abstracts using pre-specified screening criteria. The full text of any relevant articles will then be independently assessed by two reviewers. Disagreements will be resolved by discussion and, if necessary, referral to a third reviewer. The study selection process will be reported using a PRISMA flow diagram[31].

Data extraction

Data will be independently extracted by two reviewers using a standardised and piloted data extraction form. The form will be administered using the Research Electronic Data Capture (REDCap) electronic data capture tool[32]. Disagreements will be resolved by discussion and, if necessary, by referral to a third reviewer. We will choose items for extraction based on the CHARMS checklist[30], supplemented by subject-specific questions and methodological guidance. Items for extraction will include:

For peer review only - http://bmjopen.bmj.com/site/about/guidelines.xhtml

- Study characteristics [development and validation] (e.g., country, year)
- Study design [development and validation] (e.g., prospective, case control, cohort, clinical consensus)
- Patient characteristics [development and validation] (e.g., hospital ward, age, sex)
- Predicted outcome [development and validation] (e.g., survival at 24 hours, ICU admission at 24 hours)
- Model development [development] (e.g., sample size, type of model, handling of continuous variables, selection of variables, missing data, method of internal validation)
- Model presentation [development] (e.g., full regression model, simplified model, risk groups)
- Assessment of performance [development and validation] (e.g., measures of discrimination, measures of calibration)

Assessment of bias

Each article will be independently assessed by two reviewers using the Prediction model Risk of Bias ASsessment Tool (PROBAST), which was recently developed by the Cochrane Prognosis Methods Group to assess the quality and risk of bias for prediction models (due to be submitted shortly; Wolff R, Whiting, Mallett S *et al. [including author GSC]*, personal communication). PROBAST consists of 23 signalling questions within four domains (participant selection, predictors, outcome, and analysis).

For peer review only - http://bmjopen.bmj.com/site/about/guidelines.xhtml

Evidence synthesis

We will summarise the results using descriptive statistics, graphical plots, and a narrative synthesis. We do not plan to perform a quantitative synthesis of the scores or their predictive performance. However, if we identify multiple studies that evaluate the same EWS and report common performance measures, we will summarise their performance using a random-effects meta-analysis[33]. The PROBAST evaluation will be used to determine the models' risk of bias, including whether the EWSs are likely to work as intended for the hospital population of interest. The models will be classed as low, high, or unclear risk of bias.

Discussion

Although EWSs are extensively used in clinical practice, the methodology behind them remains questionable. Although not formally assessed, previous systematic reviews of EWSs have indicated that many studies suffer from a lack of quality and that few EWSs have been satisfactorily validated[9-11]. These aspects are crucial for developing a prediction model that can confidently be rolled out into clinical practice. This systematic review will bridge this important gap by examining methodological quality and external validation in detail. This systematic review is timely, as it is now nearly a decade since the last comprehensive review of EWSs, which have only existed for 20 years.

EWSs have historically been implemented as part of traditional paper observation charts. The requirement for scores to be calculated manually necessitated the use of simple scoring algorithms. Storage of data on paper has been a barrier to collection of large datasets for score derivation and validation. Digital systems are increasingly being used to record vital signs and calculate EWSs[34], offering the opportunity to be more rigorous and innovative in the development and implementation of new EWSs. The adoption of digital vital signs

For peer review only - http://bmjopen.bmj.com/site/about/guidelines.xhtml

For peer review only

charting offers an opportunity to transition away from poor quality EWSs. Our review will provide the evidence for creators of digital systems to identify which EWSs should be prioritised for implementation.

Contributors

SG, JB, TB, PW, and GSC conceived the study. SG developed the study protocol and will implement the systematic review under the supervision of GSC. SG will provide the study's statistical analysis plan and will analyse the data. SG and SK will perform the study search and SG will screen and extract the data. JB, TB, PW, and GSC will review the work. SG wrote the first protocol manuscript draft and all authors gave input into and approved the final draft of the protocol.

Funding

SG is funded by an NIHR Doctoral Fellowship (DRF-2016-09-073). JB, TB, PW and GSC are supported by the NIHR Biomedical Research Centre, Oxford. The funders have not played any role in the development of this protocol.

Competing interests

None declared

Data sharing statement

All unpublished data will be made available on request. The first author, SG, should be contacted with requests for unpublished data.

For peer review only

For peer review only - http://bmjopen.bmj.com/site/about/guidelines.xhtml

References

1. Brennan TA, Leape LL, Laird NM, et al. Incidence of Adverse Events and Negligence in Hospitalized Patients. *New England Journal of Medicine* 1991;324(6):370-76. doi: 10.1056/nejm199102073240604
2. Institute of Medicine Committee on Quality of Health Care in A. In: Kohn LT, Corrigan JM, Donaldson MS, eds. To Err is Human: Building a Safer Health System. Washington (DC): National Academies Press (US) 2000.
3. Vincent C, Neale G, Woloshynowych M. Adverse events in British hospitals: preliminary retrospective record review. *BMJ* 2001;322(7285):517-19. doi: 10.1136/bmj.322.7285.517
4. Hillman KM, Bristow PJ, Chey T, et al. Duration of life-threatening antecedents prior to intensive care admission. *Intensive Care Med* 2002;28(11):1629-34. doi: 10.1007/s00134-002-1496-y [published Online First: 2002/11/05]
5. Kause J, Smith G, Prytherch D, et al. A comparison of antecedents to cardiac arrests, deaths and emergency intensive care admissions in Australia and New Zealand, and the United Kingdom--the ACADEMIA study. *Resuscitation* 2004;62(3):275-82. doi: 10.1016/j.resuscitation.2004.05.016 [published Online First: 2004/08/25]
6. Hogan H, Healey F, Neale G, et al. Preventable deaths due to problems in care in English acute hospitals: a retrospective case record review study. *BMJ Quality & Safety* 2012
7. McQuillan P, Pilkington S, Allan A, et al. Confidential inquiry into quality of care before admission to intensive care. *Bmj* 1998;316(7148):1853-8. [published Online First: 1998/06/19]
8. Morgan RJM, Williams F, Wright MM. An early warning scoring system for detecting developing critical illness. *Clin Intensive Care* 1997;8:100.
9. Gao H, McDonnell A, Harrison DA, et al. Systematic review and evaluation of physiological track and trigger warning systems for identifying at-risk patients on the ward. *Intensive Care Medicine* 2007;33(4):667-79. doi: 10.1007/s00134-007-0532-3
10. Smith GB, Prytherch DR, Schmidt PE, et al. Review and performance evaluation of aggregate weighted 'track and trigger' systems. *Resuscitation* 2008;77(2):170-79. doi: 10.1016/j.resuscitation.2007.12.004
11. Smith MEB, Chiovaro JC, O'Neil M, et al. Early Warning System Scores for Clinical Deterioration in Hospitalized Patients: A Systematic Review. *Annals of the American Thoracic Society* 2014;11(9):1454-65. doi: 10.1513/AnnalsATS.201403-102OC
12. Excellence NIfHaC. Acutely ill adults in hospital: recognising and responding to deterioration. (NICE guideline CG50). 2007
13. Centre HSCI. Hospital Episode Statistics. Admitted Patient Care, England - 2014-15. 2015
14. Damen JAAG, Hooft L, Schuit E, et al. Prediction models for cardiovascular disease risk in the general population: systematic review. *BMJ* 2016;353 doi: 10.1136/bmj.i2416
15. Kleinrouweler CE, Cheong-See FM, Collins GS, et al. Prognostic models in obstetrics: available, but far from applicable. *Am J Obstet Gynecol* 2016;214(1):79-90.e36. doi: 10.1016/j.ajog.2015.06.013 [published Online First: 2015/06/14]
16. Bouwmeester W, Zuithoff NP, Mallett S, et al. Reporting and methods in clinical prediction research: a systematic review. *PLoS Med* 2012;9(5):1-12. doi: 10.1371/journal.pmed.1001221 [published Online First: 2012/05/26]
17. Collins GS, Mallett S, Omar O, et al. Developing risk prediction models for type 2 diabetes: a systematic review of methodology and reporting. *BMC Med* 2011;9:103. doi: 10.1186/1741-7015-9-103 [published Online First: 2011/09/10]
18. Collins GS, de Groot JA, Dutton S, et al. External validation of multivariable prediction models: a systematic review of methodological conduct and reporting. *BMC Med Res Methodol* 2014;14:40. doi: 10.1186/1471-2288-14-40 [published Online First: 2014/03/22]
19. Mallett S, Royston P, Waters R, et al. Reporting performance of prognostic models in cancer: a review. *BMC Med* 2010;8:21. doi: 10.1186/1741-7015-8-21 [published Online First: 2010/04/01]

For peer review only - http://bmjopen.bmj.com/site/about/guidelines.xhtml

20. NCEPOD. Time to Intervene? A review of patients who underwent cardiopulmonary rescuscitation as a result of an in-hospital cardiorespiratory arrest. 2012
21. Kyriacos U, Jelsma J, Jordan S. Monitoring vital signs using early warning scoring systems: a review of the literature. *Journal of nursing management* 2011;19(3):311-30. doi: 10.1111/j.1365-2834.2011.01246.x [published Online First: 2011/04/22]
22. Alam N, Hobbelink EL, van Tienhoven AJ, et al. The impact of the use of the Early Warning Score (EWS) on patient outcomes: a systematic review. *Resuscitation* 2014;85(5):587-94. doi: 10.1016/j.resuscitation.2014.01.013 [published Online First: 2014/01/29]
23. McGaughey J, Alderdice F, Fowler R, et al. Outreach and Early Warning Systems (EWS) for the prevention of Intensive Care admission and death of critically ill adult patients on general hospital wards. *Cochrane Database of Systematic Reviews* 2007(3) doi: 10.1002/14651858.CD005529.pub2
24. Collins GS, Reitsma JB, Altman DG, et al. Transparent reporting of a multivariable prediction model for individual prognosis or diagnosis (tripod): The tripod statement. *Annals of Internal Medicine* 2015;162(1):55-63. doi: 10.7326/M14-0697
25. Moons KG, Altman DG, Reitsma JB, et al. Transparent Reporting of a multivariable prediction model for Individual Prognosis or Diagnosis (TRIPOD): explanation and elaboration. *Ann Intern Med* 2015;162(1):W1-73. doi: 10.7326/m14-0698 [published Online First: 2015/01/07]
26. Moons KG, Kengne AP, Woodward M, et al. Risk prediction models: I. Development, internal validation, and assessing the incremental value of a new (bio)marker. *Heart* 2012;98(9):683-90. doi: 10.1136/heartjnl-2011-301246 [published Online First: 2012/03/09]
27. Steyerberg EW, Moons KG, van der Windt DA, et al. Prognosis Research Strategy (PROGRESS) 3: prognostic model research. *PLoS Med* 2013;10(2):e1001381. doi: 10.1371/journal.pmed.1001381 [published Online First: 2013/02/09]
28. Steyerberg EW, Vergouwe Y. Towards better clinical prediction models: seven steps for development and an ABCD for validation. *Eur Heart J* 2014;35(29):1925-31. doi: 10.1093/eurheartj/ehu207 [published Online First: 2014/06/06]
29. Justice AC, Covinsky KE, Berlin JA. Assessing the generalizability of prognostic information. *Ann Intern Med* 1999;130(6):515-24. [published Online First: 1999/03/13]
30. Moons KG, de Groot JA, Bouwmeester W, et al. Critical appraisal and data extraction for systematic reviews of prediction modelling studies: the CHARMS checklist. *PLoS Med* 2014;11(10):e1001744. doi: 10.1371/journal.pmed.1001744 [published Online First: 2014/10/15]
31. Moher D, Liberati A, Tetzlaff J, et al. Preferred Reporting Items for Systematic Reviews and Meta-Analyses: The PRISMA Statement. *PLOS Medicine* 2009;6(7):e1000097. doi: 10.1371/journal.pmed.1000097
32. Harris PA, Taylor R, Thielke R, et al. Research Electronic Data Capture (REDCap) - A metadata-driven methodology and workflow process for providing translational research informatics support. *Journal of biomedical informatics* 2009;42(2):377-81. doi: 10.1016/j.jbi.2008.08.010
33. Debray TP, Damen JA, Snell KI, et al. A guide to systematic review and meta-analysis of prediction model performance. *Bmj* 2017;356:i6460. doi: 10.1136/bmj.i6460 [published Online First: 2017/01/07]
34. Wong D, Bonnici T, Knight J, et al. SEND: a system for electronic notification and documentation of vital sign observations. *BMC Medical Informatics and Decision Making* 2015;15:68. doi: 10.1186/s12911-015-0186-y

For peer review only - http://bmjopen.bmj.com/site/about/guidelines.xhtml

MEDLINE search strategy

Database version: Ovid MEDLINE(R) Epub Ahead of Print, In-Process & Other Non-Indexed Citations, Ovid MEDLINE(R) Daily and Ovid MEDLINE(R) 1946 to Present

1. ((National or VitalPAC or Modified or Centile or standard$) adj1 Early adj1 Warning adj1 Scor$).ti,ab.
2. (Reading adj1 Modified adj1 Early adj1 Warning adj1 Score).ti,ab.
3. (Cardiac adj1 Arrest adj1 Risk adj1 Triage).ti,ab.
4. (Assessment adj1 Score adj2 Sick adj1 patient adj1 Identification adj2 Step-up adj2 Treatment).ti,ab.
5. (Targeted adj1 Real adj1 Time adj1 Early adj1 Warning adj1 Score).ti,ab.
6. (Dutch adj1 Early adj1 Nurse adj1 Worry adj1 Indicator adj1 Score).ti,ab.
7. (Decision adj1 Tree adj1 Early adj1 Warning adj1 Score).ti,ab.
8. (Advanced adj1 Alert adj1 Monitor).ti,ab.
9. (Chronic adj1 Respiratory adj1 Early adj1 Warning adj1 Score).ti,ab.
10. (early adj1 warning adj1 (scor$ or system$)).ti,ab.
11. (track adj2 trigger adj2 (scor$ or system$)).ti,ab.
12. (physiological adj1 scoring adj1 system$).ti,ab.
13. (worry adj1 indicator adj1 scor$).ti,ab.
14. (physiological adj1 observation adj1 track adj2 trigger adj1 (scor$ or system$)).ti,ab.
15. (patient adj2 risk adj2 scoring adj1 system$).ti,ab.
16. (patient adj2 risk adj1 trigger adj1 scoring adj1 system).ti,ab.
17. (early adj1 detection adj2 patients adj2 risk).ti,ab.
18. Early Warning Score.kw.
19. Track-and-Trigger.kw.
20. "track and trigger".kw.
21. or/1-20
22. Predictive Value of Tests/
23. Monitoring, Physiologic/
24. Nursing Assessment/mt
25. Nursing Assessment/st
26. Nursing Assessment/sn
27. Severity of Illness Index/
28. Health Status Indicators/
29. Point-of-Care-Systems/

For peer review only

30. (NEWS or ViEWS or CART or SEWS or CREWS or PAR or PART or PSS or AAM).ti,ab.

31. OR/22-30

32. ((early or risk or warn$ or alert$ or track$ or trigger) adj2 (scor$ or system or systems)).ti,ab.

33. 31 AND 32

34. (MEWS or R-MEWS or eCART or CEWS or TREWScore or DENWIS or DTEWS or DMEWS or POTTS or PAR-T or ViEWS-L).ti,ab.

35. (develop$ or design$ or creat$ or build$ or contruct$ or validat$).ti,ab.

36. Validation Studies.pt.

37. 35 OR 36

38. 33 AND 37

39. 34 AND 37

40. 38 OR 39

41. 21 OR 40

42. ((child OR infant OR pediatrics) NOT adult).sh.

43. 41 NOT 42

For peer review only

1

PRISMA-P 2015 Checklist

Section/topic	#	Checklist item	Information reported Yes	Information reported No	Page number
**ADMINISTRATIVE INFORMATION**					
**Title**					
Identification	1a	Identify the report as a protocol of a systematic review	☒	☐	1
Update	1b	If the protocol is for an update of a previous systematic review, identify as such	☒	☐	NA
**Registration**	2	If registered, provide the name of the registry (e.g., PROSPERO) and registration number in the Abstract	☒	☐	2 & 7
**Authors**					
Contact	3a	Provide name, institutional affiliation, and e-mail address of all protocol authors; provide physical mailing address of corresponding author	☒	☐	1
Contributions	3b	Describe contributions of protocol authors and identify the guarantor of the review	☒	☐	11 & 12
**Amendments**	4	If the protocol represents an amendment of a previously completed or published protocol, identify as such and list changes; otherwise, state plan for documenting important protocol amendments	☒	☐	NA
**Support**					
Sources	5a	Indicate sources of financial or other support for the review	☒	☐	12
Sponsor	5b	Provide name for the review funder and/or sponsor	☒	☐	12
Role of sponsor/funder	5c	Describe roles of funder(s), sponsor(s), and/or institution(s), if any, in developing the protocol	☒	☐	12
**INTRODUCTION**					
**Rationale**	6	Describe the rationale for the review in the context of what is already known	☒	☐	3 & 4
**Objectives**	7	Provide an explicit statement of the question(s) the review will address with reference to participants, interventions, comparators, and outcomes (PICO)	☒	☐	7
**METHODS**					
**Eligibility criteria**	8	Specify the study characteristics (e.g., PICO, study design, setting, time frame) and report characteristics (e.g., years considered, language, publication status) to be used as criteria for eligibility for the review	☒	☐	8

For peer review only

2

Section/topic	#	Checklist item	Information reported Yes	Information reported No	Page number
**Information sources**	9	Describe all intended information sources (e.g., electronic databases, contact with study authors, trial registers, or other grey literature sources) with planned dates of coverage	☒	☐	8
**Search strategy**	10	Present draft of search strategy to be used for at least one electronic database, including planned limits, such that it could be repeated	☒	☐	Page 15 (Appendix A)
STUDY RECORDS					
Data management	11a	Describe the mechanism(s) that will be used to manage records and data throughout the review	☒	☐	9
Selection process	11b	State the process that will be used for selecting studies (e.g., two independent reviewers) through each phase of the review (i.e., screening, eligibility, and inclusion in meta-analysis)	☒	☐	9
Data collection process	11c	Describe planned method of extracting data from reports (e.g., piloting forms, done independently, in duplicate), any processes for obtaining and confirming data from investigators	☒	☐	9
**Data items**	12	List and define all variables for which data will be sought (e.g., PICO items, funding sources), any pre-planned data assumptions and simplifications	☒	☐	9
**Outcomes and prioritization**	13	List and define all outcomes for which data will be sought, including prioritization of main and additional outcomes, with rationale	☒	☐	9
**Risk of bias in individual studies**	14	Describe anticipated methods for assessing risk of bias of individual studies, including whether this will be done at the outcome or study level, or both; state how this information will be used in data synthesis	☒	☐	10
DATA					
**Synthesis**	15a	Describe criteria under which study data will be quantitatively synthesized	☒	☐	10
	15b	If data are appropriate for quantitative synthesis, describe planned summary measures, methods of handling data, and methods of combining data from studies, including any planned exploration of consistency (e.g., I^2, Kendall's tau)	☒	☐	10
	15c	Describe any proposed additional analyses (e.g., sensitivity or subgroup analyses, meta-regression)	☒	☐	NA
	15d	If quantitative synthesis is not appropriate, describe the type of summary planned	☒	☐	10
**Meta-bias(es)**	16	Specify any planned assessment of meta-bias(es) (e.g., publication bias across studies, selective reporting within studies)	☒	☐	NA
**Confidence in cumulative evidence**	17	Describe how the strength of the body of evidence will be assessed (e.g., GRADE)	☒	☐	NA

For peer review only
